# Supplementary material for: Anti-IL-6 Versus Anti-IL-6R Blocking Antibodies to Treat Acute Ebola Infection in BALB/c Mice: Potential Implications for Treating Cytokine Release Syndrome
Source: Front Pharmacol. 2020 Sep 23;11:574703. doi: 10.3389/fphar.2020.574703 (PMC7538647; doi:10.3389/fphar.2020.574703)
Supplement: Supplementary file 1 [file DataSheet_1.pdf]

Anti-IL-6 *versus* Anti-IL-6R Blocking Antibodies to Treat Acute Ebola Infection in BALB/c Mice: Potential Implications for Treating Cytokine Release Syndrome

1 SUPPLEMENTARY TABLES AND FIGURES

1.1 Figures

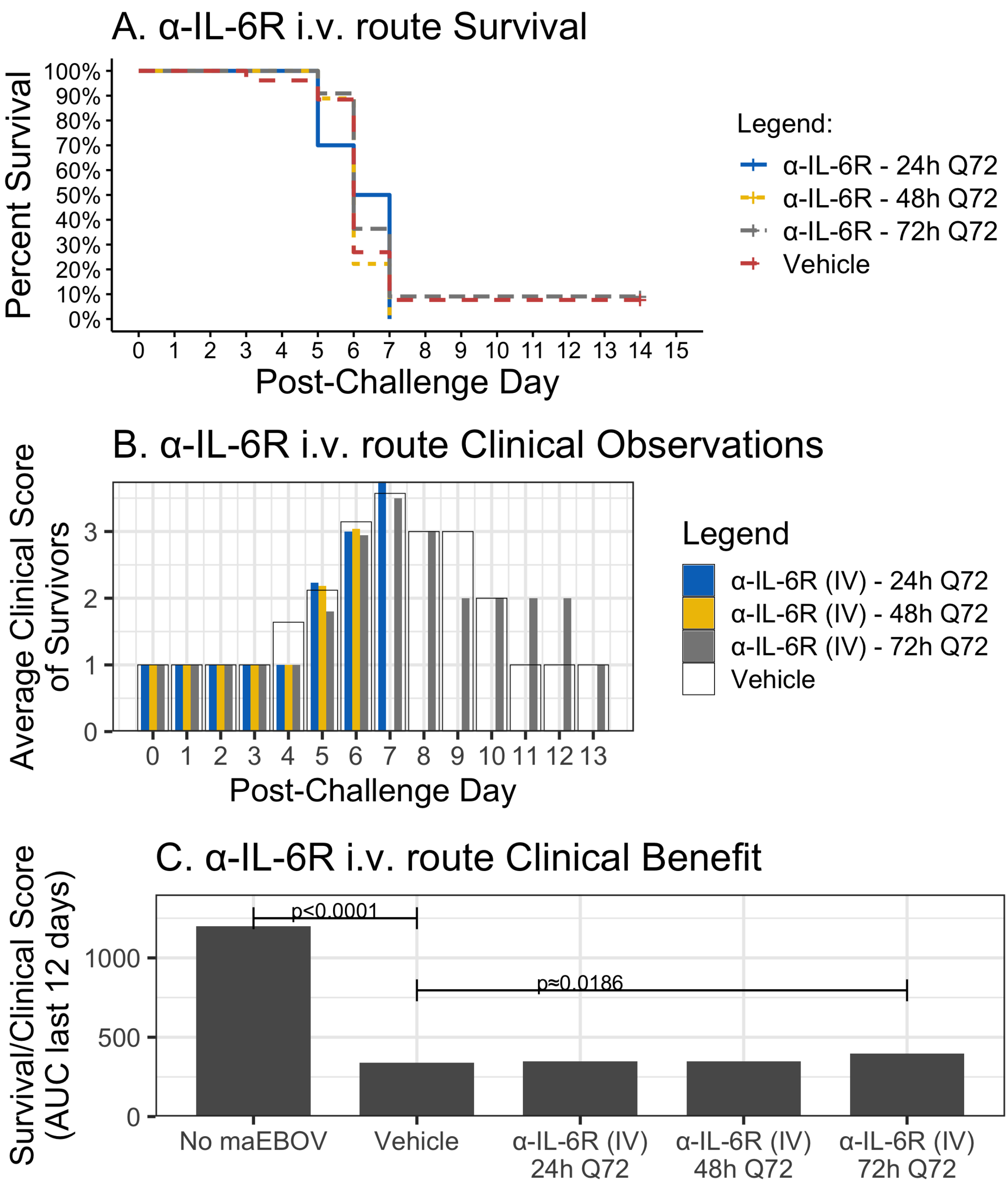

Figure S1: Survival, Clinical Scores and AUC Survival/Clinical score for one i.v. dose α-IL-6R. (a) Kaplan Meier Plot of mouse survival receiving one i.v. dose of α-IL-6R 24 post maEBOV challenge. The curves were not significantly different by Log-rank (Mantel-Cox) testing. (b) Average Clinical scores for surviving mice receiving one i.v. dose α-IL-6R 24 post maEBOV. The SEM were $<$ 10% of the mean for clinical scores. (c) A composite benefit metric was calculated as the AUC for the last 12 days of the quotient of survival and clinical score. The AUC for a group of healthy untreated mice (for example, 100% survival with a clinical score of 1 (healthy) observed for twelve days would be calculated as 1200).

1.2 Tables

Clinical Score	Description of Animal
1	Healthy
2	Lethargic and/or ruffled fur<br>(triggers a second observation)
3	Ruffled fur, lethargic and hunched posture, orbital tightening<br>(triggers a third observation)
4	Ruffled fur, lethargic, hunched posture, orbital tightening<br>reluctance to move when stimulated, paralysis or greater than 20% weight loss.
(no score)	Deceased

Table S1. Clinical score indices used to record morbidity in study animals.

Group	N	Test Article	Dosing Regimen
1	6	Dilution Buffer Vehicle (i.v.)	One dose 24 hours post challenge<br>no subsequent doses given
2	10	α-IL-6R mAb (100 ug i.v.)	One dose 24 hours post challenge<br>no subsequent doses given
3	10	α-IL-6R mAb (100 ug i.v.)	One dose 48 hours post challenge<br>no subsequent doses given
4	10	α-IL-6R mAb (100 ug i.v.)	One dose 72 hours post challenge<br>no subsequent doses given

Table S2. i.v. delivery experiment design. All mice were challenged with 100 plaque forming units of maEBOV via intraperitoneal injection. Antibody treatments were given in a volume of 100 uL. Group 1 consisted of three male and three female mice. Groups 2-4 were comprised of 10 mice (5M/5F).

Group	N	Test Article	Dosing Regimen
1	10	Dilution Buffer Vehicle (i.p.)	First dose 24 hours post challenge subsequent doses Q72 hours
2	10	α-mouse-IL-6 mAb (400 ug i.p.)	First dose 24 hours post challenge subsequent doses Q72 hours
3	10	α-mouse-IL-6 mAb (400 ug i.p.)	First dose 48 hours post challenge subsequent doses Q72 hours
4	10	α-mouse-IL-6 mAb (400 ug i.p.)	First dose 72 hours post challenge subsequent doses Q72 hours
5	10	α-mouse-IL-6R mAb (400 ug i.p.)	First dose 24 hours post challenge subsequent doses Q72 hours
6	10	α-mouse-IL-6R mAb (400 ug i.p.)	First dose 48 hours post challenge subsequent doses Q72 hours
7	10	α-mouse-IL-6R mAb (400 ug i.p.)	First dose 72 hours post challenge subsequent doses Q72 hours

Table S3. First intraperitoneal delivery experiment design. All mice were challenged with 100 plaque forming units of maEBOV via intraperitoneal injection. Antibody treatments were given in a volume of 100uL. All groups were comprised of 10 mice (5M/5F).

Group	N	Test Article	Dosing Regimen
1	20	Dilution Buffer Vehicle (i.p)	First dose 24 hours post challenge no subsequent doses
2	20	α-mouse-IL-6 mAb (400 ug i.p.)	First dose 24 hours post challenge no subsequent doses

Table S4. Second i.p. delivery experiment design. All mice were challenged with 100 plaque forming units of maEBOV via intraperitoneal injection. Antibody treatments were given in a volume of 100 uL. All groups were comprised of 10 mice (5M/5F).

Antibody	Route	Dose (ug)	Dose (mg/Kg)	$T_{1/2}(h)$	$K_{el}(h^{-1})$	$K_a(h^{-1})$	$V_d(L/Kg)$	F
α-IL-6R	IV	100	5	223	0.0031	-	0.05	1
α-IL-6R	IP	400	20	223	0.0031	0.5	0.05	0.5
α-IL-6	IP	400	20	57	0.0122	0.5	0.05	0.5

Table S5. Pharmacokinetic parameters predicted based on literature values for the monoclonal antibodies used for the study are shown. $T_{1/2}$ is the terminal half life. Although antibody blood levels were not measured, this allowed simulated PK profiles to be created as shown in Figure 4 of the manuscript.
